# LXR signaling pathways link cholesterol metabolism with risk for prediabetes and diabetes

**DOI:** 10.1172/JCI173278

**Published:** 2024-05-15

**Authors:** Jingzhong Ding, Anh Tram Nguyen, Kurt Lohman, Michael T. Hensley, Daniel Parker, Li Hou, Jackson Taylor, Deepak Voora, Janet K. Sawyer, Elena Boudyguina, Michael P. Bancks, Alain Bertoni, James S. Pankow, Jerome I. Rotter, Mark O. Goodarzi, Russell P. Tracy, David M. Murdoch, Daniel Duprez, Stephen S. Rich, Bruce M. Psaty, David Siscovick, Christopher B. Newgard, David Herrington, Ina Hoeschele, Steven Shea, James H. Stein, Manesh Patel, Wendy Post, David Jacobs, John S. Parks, Yongmei Liu

**Affiliations:** 1 Department of Internal Medicine, Wake Forest School of Medicine, Winston-Salem, North Carolina, USA.; 2 Department of Medicine, Division of Cardiology, and; 3 Department of Medicine, Division of Geriatrics, Duke University, Durham, North Carolina, USA.; 4 Department of Biological, Geological, and Environmental Sciences, Cleveland State University, Cleveland, Ohio, USA.;; 5 Department of Epidemiology and Prevention, Wake Forest School of Medicine, Winston-Salem, North Carolina, USA; 6 Division of Epidemiology and Community Health, University of Minnesota, Minneapolis, Minnesota, USA.; 7 The Institute for Translational Genomics and Population Sciences, Department of Pediatrics, The Lundquist Institute for Biomedical Innovation at Harbor-UCLA Medical Center, Torrance, California, USA; 8 Division of Endocrinology, Diabetes and Metabolism, Cedars-Sinai Medical Center, Los Angeles, California, USA.; 9 Department of Pathology and Laboratory Medicine, University of Vermont, Burlington, Vermont, USA.; 10 Department of Medicine, Division of Pulmonary, Allergy, and Critical Care Medicine, Duke University, Durham, North Carolina, USA.; 11 Cardiovascular Division, Department of Medicine, University of Minnesota, Minneapolis, Minnesota, USA.; 12 Center for Public Health Genomics, University of Virginia, Charlottesville, Virginia, USA.; 13 Cardiovascular Health Research Unit, Departments of Medicine, Epidemiology, and Health Systems and Population Health, University of Washington, Seattle, Washington, USA.; 14 New York Academy of Medicine, New York, New York, USA.; 15 Department of Pharmacology and Cancer Biology, Duke University, Durham, North Carolina, USA.; 16 Fralin Life Sciences Institute, Virginia Tech, Blacksburg, Virginia, USA.; 17 Department of Medicine, Columbia University, New York, New York, USA.; 18 School of Medicine and Public Health, University of Wisconsin, Madison, Wisconsin, USA.; 19 Division of Cardiology, Department of Medicine, Johns Hopkins University, Baltimore, Maryland, USA.

**Keywords:** Metabolism, Cholesterol, Diabetes, Expression profiling

## Abstract

**BACKGROUND:**

Preclinical studies suggest that cholesterol accumulation leads to insulin resistance. We previously reported that alterations in a monocyte cholesterol metabolism transcriptional network (CMTN) — suggestive of cellular cholesterol accumulation — were cross-sectionally associated with obesity and type 2 diabetes (T2D). Here, we sought to determine whether the CMTN alterations independently predict incident prediabetes/T2D risk, and correlate with cellular cholesterol accumulation.

**METHODS:**

Monocyte mRNA expression of 11 CMTN genes was quantified among 934 Multi-Ethnic Study of Atherosclerosis (MESA) participants free of prediabetes/T2D; cellular cholesterol was measured in a subset of 24 monocyte samples.

**RESULTS:**

During a median 6-year follow-up, lower expression of 3 highly correlated LXR target genes — *ABCG1* and *ABCA1* (cholesterol efflux) and *MYLIP* (cholesterol uptake suppression) — and not other CMTN genes, was significantly associated with higher risk of incident prediabetes/T2D. Lower expression of the LXR target genes correlated with higher cellular cholesterol levels (e.g., 47% of variance in cellular total cholesterol explained by *ABCG1* expression). Further, adding the LXR target genes to overweight/obesity and other known predictors significantly improved prediction of incident prediabetes/T2D.

**CONCLUSION:**

These data suggest that the aberrant LXR/ABCG1-ABCA1-MYLIP pathway (LAAMP) is a major T2D risk factor and support a potential role for aberrant LAAMP and cellular cholesterol accumulation in diabetogenesis.

**FUNDING:**

The MESA Epigenomics and Transcriptomics Studies were funded by NIH grants 1R01HL101250, 1RF1AG054474, R01HL126477, R01DK101921, and R01HL135009. This work was supported by funding from NIDDK R01DK103531 and NHLBI R01HL119962.

## Abstract 2

The dysregulated LXRβ/ABCG1-ABCA1-MYLIP signaling pathway, coincident with cellular cholesterol accumulation, is a major risk factor for type 2 diabetes.

## Introduction

While hypercholesterolemia is a common feature of patients with type 2 diabetes (T2D), its role in the pathogenesis of this disease is not well established. The negative impact of cellular cholesterol accumulation on glucose metabolism has been recently reported in preclinical studies (1–5), although its role in human T2D remains unclear. Recently, using transcriptomic profiling in monocyte samples from Multi-Ethnic Study of Atherosclerosis (MESA) participants, we reported that alterations of a cholesterol metabolism transcriptional network (CMTN) were more frequently observed in individuals with obesity, chronic inflammation, prevalent T2D, or atherosclerosis burden (6). Coordinated gene expression changes of the 11 CMTN members are expected to increase overall cellular cholesterol content through downregulation of cholesterol efflux (↓*ABCG1*, *ABCA1*), upregulation of cholesterol synthesis (↑*SCD*, *SQLE*, *HMGCS1*, *FDFT1*, *FADS1*, *CYP51A1*, and *SC4MOL*), and upregulation of sterol uptake (↑*LDLR*, ↓*MYLIP*). However, whether aberrant cholesterol metabolism contributes to the development of T2D in humans could not be inferred in the cross-sectional study given that chronic elevated glucose can lead to disturbances in cholesterol metabolism (7, 8).

In a large MESA community-based population cohort free of prediabetes and T2D at baseline, we sought to examine predictive effects of alterations in monocyte CMTN on the incidence of prediabetes and diabetes. To better understand the cellular effect of dysregulated CMTN, we assessed correlations between the altered CMTN and cellular cholesterol accumulation.

## Results

**Figure 1:**
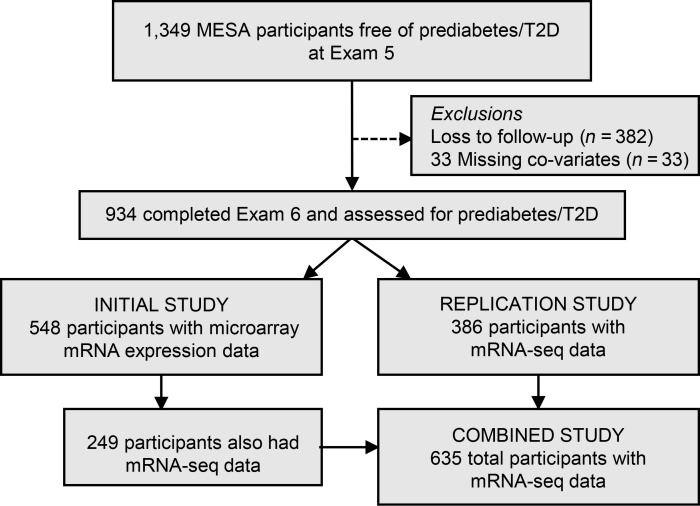
Overview of the study design and baseline characteristics. Prospective analyses to examine predictive effects of the CMTN on the incidence of prediabetes/T2D over a 6-year follow-up among 1,349 participants with euglycemia at Exam 5, using subsets of samples.

The Multi-Ethnic Study of Atherosclerosis (MESA) was designed to investigate the progression of subclinical cardiovascular disease in a community-based cohort (9). Of 1,349 MESA participants with euglycemia (free of prediabetes/T2D; see Supplemental Table 1; supplemental material available online with this article; https://doi.org/10.1172/JCI173278DS1), who completed Exam 5 between 2010 and 2012 (designated as the “baseline” examination period for the present investigation), 967 completed Exam 6 follow-up to assess for prediabetes/T2D (Figure 1). Of those, 33 had missing covariates, leaving 934 participants for monocyte transcriptomic profiling and subsequent analyses. An Initial Study was conducted for a random subset of 548 MESA participants using microarray technology for transcriptomic profiling. The Replication Study consisted of the remaining 386 MESA participants using sequencing technology (mRNA-Seq). To obtain more precise parameter estimates with a larger sample size, a total of 635 monocyte mRNA-Seq samples, which includes a subset of 249 participants from the Initial Study and all 386 participants from the Replication Study, were analyzed together and designated as a Combined Study (Figure 1).

### Initial Study.

**Figure 2:**
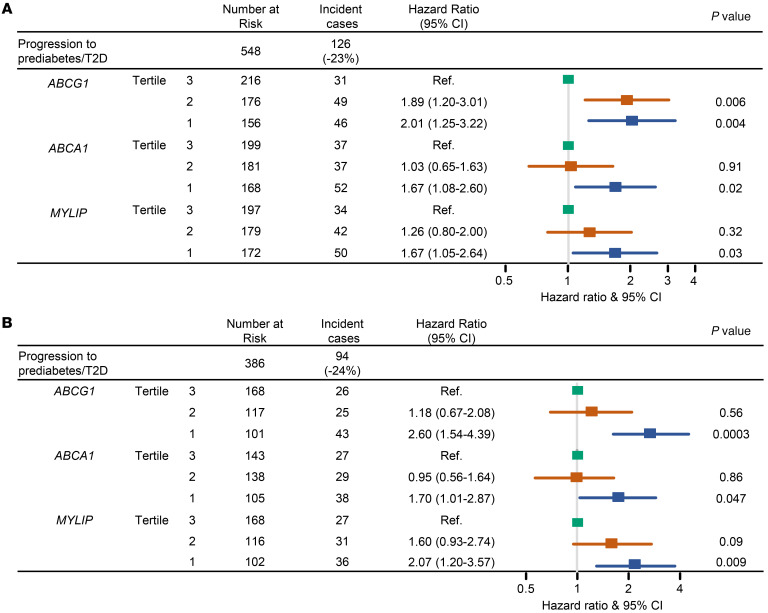
Expression of LXRα target genes predicts risk of prediabetes and T2D over a 6-year follow-up. MESA participants with lower expression of LXRα target genes, *ABCA1*, *ABCG1*, and *MYLIP* (lowest tertile), in 2 independent sub-studies, the Initial Study (**A**) and the Replication Study (**B**), were more likely to develop prediabetes/T2D compared with those with greater expression (highest tertile). Cox proportional hazards regression models were used, adjusting for age, sex, race and ethnicity, cigarette smoking, physical activity level, BMI, triglycerides, HDL-cholesterol, and systolic blood pressure (SBP) in the full model. The *x* axis is in logarithmic scale.

Of 548 with euglycemia, there were 126 incident cases of prediabetes (*n*_case_ = 106) or T2D (*n*_case_ = 20) during a median 6-year follow-up (Figure 2A). In the full model with each of the genes analyzed individually, adjusting for age, sex, race and ethnicity, cigarette smoking, physical activity level, BMI, triglycerides, systolic blood pressure, and plasma HDL-cholesterol (HDL-C), risk for prediabetes/T2D was higher among MESA participants in the lowest tertile of expression of 3 coexpressed LXR target genes (10–12) — *ABCG1*, *ABCA1*, and *MYLIP* (pairwise correlation between expression of the 3 genes ranges from 0.47 to 0.71; Supplemental Table 2). Specifically, hazard ratios (HRs) for the first versus third tertile were 2.01 (95% CI, 1.25–3.22) for *ABCG1*, 1.67 (95% CI, 1.08–2.60) for *ABCA1*, and 1.67 (95% CI, 1.05–2.64) for *MYLIP* (also known as IDOL [inducible degrader of the LDL receptor]).

### Replication Study.

Of 386 with euglycemia, 94 developed incident prediabetes (*n*_case_ = 81) or T2D (*n*_case_ = 13) during a median 6-year follow-up. Using the same modeling approach as the Initial Study, similar results were observed in the Replication Study (Figure 2B and Supplemental Table 2), with *ABCG1* associations being most substantial (HR for the first vs. third tertile, 2.60; 95% CI, 1.54–4.39), followed by *MYLIP* and *ABCA1* (HR for the first vs. third tertile, 2.07 and 1.70; 95% CI, 1.20–3.57 and 1.01–2.87, respectively). There appeared to be a graded inverse association between increasing tertiles of *ABCG1* and *MYLIP* expression and the risk of incident prediabetes/T2D in both the Initial and the Replication Study.

### Combined Study.

To capture the independent sets of cholesterol metabolism pathways, a principal component analysis (PCA) was performed for the data on the 11 CMTN gene members. PCA showed 2 main principal components (PCs) in CMTN, PC1_CMTN_ (the first PC of the CMTN) and PC2_CMTN_ (second PC), which explained 43% and 20% of the CMTN variation, respectively (Supplemental Figure 1; *n* = 635). PC1_CMTN_ was strongly and positively correlated with SREBP2 (1 of 2 sterol-responsive transcription factors) target genes (cholesterol synthesis, e.g., *SQLE*, and uptake genes) and weakly and inversely correlated with the 3 LXR target genes (Table 1). Thus, PC1_CMTN_ is predicted to be positively correlated with cellular cholesterol levels. PC2_CMTN_ was strongly and positively correlated with the LXR target genes, *ABCG1* (*r* = 0.85), *ABCA1* (*r* = 0.85), and *MYLIP* (*r* = 0.65) (Table 1). Thus, PC2_CMTN_ is predicted to be inversely correlated with cellular cholesterol levels.

Expression of the 3 LXR target genes, *ABCG1*, *ABCA1*, and *MYLIP*, was inversely associated with risk of prediabetes/T2D, with *ABCG1* associations (HR, 1.33 per 1-SD decrease; 95% CI, 1.14–1.56; *P* = 3.63 × 10^–4^ in the full model; Table 2) being similar to that of *MYLIP*. As expected, PC2_CMTN_, which strongly and positively correlated with expression of the 3 LXR target genes, was also inversely associated with incident prediabetes/T2D (HR, 1.28 per 1-SD decrease; 95% CI, 1.13–1.45; *P* = 8.67 × 10^–5^ in the full model), although the association was slightly weaker than that of *ABCG1/MYLIP*. These associations were independent of plasma total or LDL-cholesterol levels, comorbidities such as prevalent CVD, and statin use (data not shown). Further stratified analyses showed that these associations were consistent across sex and racial and ethnic subgroups (except among Hispanic participants, which was the smallest subgroup, *n* = 105; Supplemental Table 3). The effects in the younger subgroup (<70 years) were slightly stronger than those in the older subgroup (Supplemental Table 3); the age by PC2_CMTN_ interaction term was marginally significant (*P* = 0.05 in the full model). PC1_CMTN_, as well as the 8 individual PC1_CMTN_-correlated genes, did not associate with incident prediabetes/T2D (Table 1 and Supplemental Table 4).

Given that *ABCG1*, *ABCA1*, and *MYLIP* are target genes of LXRα (primarily expressed in liver, intestine, and kidney) and LXRβ (expressed ubiquitously; ref. 13), expression of *NR1H3* and *NR1H2*, which encode LXRα and LXRβ, respectively, was also examined. In the Combined Study, HRs of incident prediabetes/T2D for *NR1H2* were 1.40 (95% CI, 0.93–2.12) for the second versus third tertile and 1.53 (95% CI, 1.03–2.28) for the first versus third tertile in the full model. The association of *NR1H3* (encoding LXRα) and lipogenic LXR target genes (14), e.g., *SREBF1* (encoding SREBP1, a lipogenic transcription factor), with incident prediabetes/T2D was not significant.

**Figure 3:**
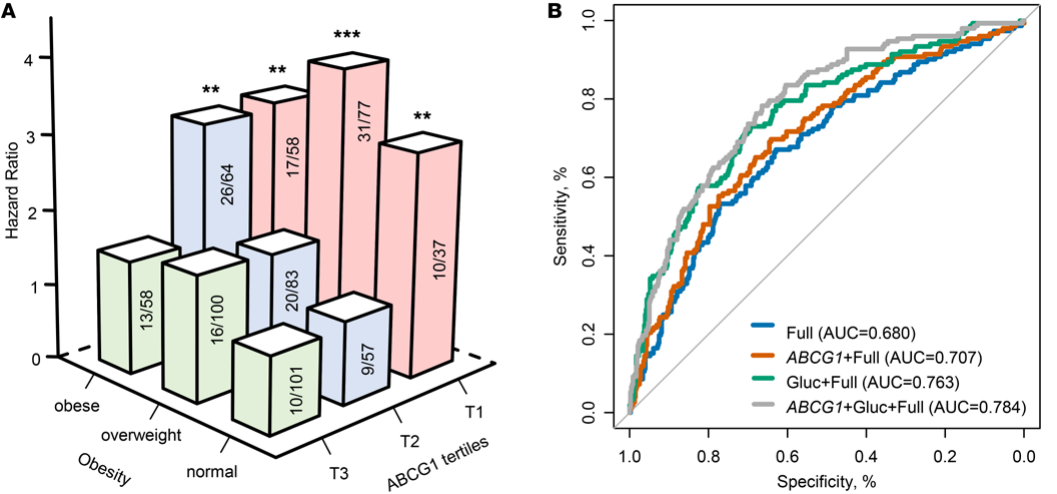
Predictive effects of *ABCG1* expression for incident prediabetes/T2D among 635 participants with euglycemia. (**A**) Bar plot for hazard ratio of prediabetes/diabetes according to baseline obesity status and tertiles of *ABCG1* expression, adjusting for age, sex, race and ethnicity, cigarette smoking, physical activity level, triglycerides, HDL-cholesterol, and SBP. Number of cases/number at risk is displayed for each cell. **P < 0.01, ***P < 0.001. (**B**) AUC–receiver operating characteristic curves for 4 models with or without *ABCG1* expression. The AUC is Harrell’s C statistic from a Cox regression model. The full model includes BMI, along with age, sex, race and ethnicity, cigarette smoking, physical activity level, triglycerides, HDL-cholesterol, and SBP. Gluc, fasting glucose.

The three LXR target genes were weakly correlated with several characteristics — inversely with age, African American race, BMI, triglycerides, and fasting glucose, while positively with HDL-C and LDL-C levels (shown in Supplemental Table 5 for *ABCG1* expression). Adjustment for BMI did not affect their associations with incident prediabetes/T2D, and their interaction term with BMI was not significant. Mediation analyses were performed to study whether their associations mediate the association of BMI with incident prediabetes/T2D. Indirect effects of BMI on prediabetes/T2D risk mediated through *ABCG1* expression were significant (odds ratio via *ABCG1* expression: 1.04 per 1-SD increase in BMI; 95% CI, 1.01–1.07; *P* = 0.005). Expression of *ABCG1* explained 24% (*P* = 0.04) of the effect of BMI on prediabetes/T2D risk. The combinatorial effects of overweight/obesity and *ABCG1* expression were evaluated further (Figure 3A). The risk of prediabetes/T2D for overweight/obese individuals was highest when they also had lower levels of *ABCG1* expression compared with normal-weight individuals with the third tertile of *ABCG1* expression (HR for individuals with the first tertile of *ABCG1* expression and overweight: 3.83 *P* = 0.0002; or obesity: 3.12, *P* = 0.003). Tertile 1 (vs. tertile 3) for *ABCG1* expression was associated with incident prediabetes/T2D even in those with normal weight.

Risk prediction models were also developed. The model that included age, sex, race and ethnicity, and *ABCG1* expression had a C statistic of 0.672, compared with a C statistic of 0.680 in the full model that included BMI, as well as age, sex, race and ethnicity, cigarette smoking, physical activity level, triglycerides, HDL-C, and systolic blood pressure. When *ABCG1* expression was added to the full model, the C statistic increased to 0.707 (Figure 3B). The model improvement with *ABCG1* expression added was statistically significant (likelihood ratio *P* = 0.0003). The findings were similar when BMI was categorized as normal, overweigh, or obese. Adding *ABCG1* expression tertiles to the full model, including the overweight/obese categories, significantly improved classification accuracy (net reclassification improvement index [NRI], 0.42; 95% CI, 0.21–0.63). Compared with the full model plus fasting glucose, the addition of *ABCG1* expression improved the discrimination and reclassification indexes for prediabetes/T2D, including the C statistic (from 0.763 to 0.784), likelihood ratio test (*P* = 0.0001), and NRI (0.39; 95% CI, 0.18–0.63; Figure 3B).

### Association of CMTN gene expression with cellular cholesterol.

**Figure 4:**
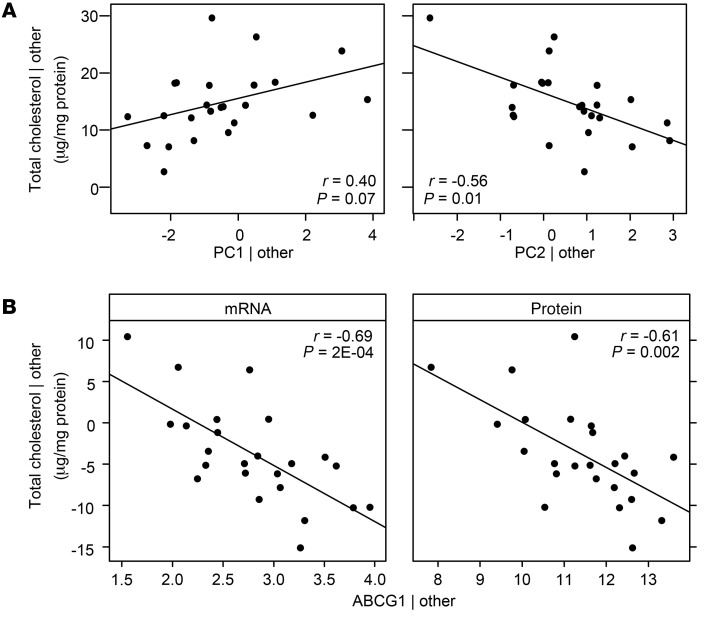
CMTN associations with monocyte total cellular cholesterol. Added variable plots with adjustment of age, sex, and race and ethnicity are shown for correlation of 2 top CMTN principal components, PC1_CMTN_ and PC2_CMTN_ (**A**), or *ABCG1* mRNA and protein expression (**B**), with total cholesterol in primary monocytes from a subset of 24 randomly selected participants. Partial Spearman’s *r* is reported. Total cholesterol was measured using gas chromatography and normalized to protein levels (Bradford assay). “Other” indicates age, sex, race and ethnicity, and batch effect. ABCG1 protein levels were measured by Western blot analysis.

A cross-sectional analysis was performed on a subset of 24 randomly selected participants who had their monocytes purified and measured for both mRNA-Seq and cellular cholesterol between 2016 and 2018 (Exam 6). Consistent with what was predicted in the Combined Study, PC1_CMTN_ (*r* = 0.40) was positively correlated while PC2_CMTN_ (*r* = –0.56) and *ABCG1* (*r* = –0.69) were inversely correlated with cellular total cholesterol (Figure 4, A and B, and Supplemental Table 6 for other CMTN members). PC1_CMTN_, PC2_CMTN_, and *ABCG1* explained 14%, 28%, and 47% of the variance in cellular total cholesterol levels, respectively. In contrast, cellular cholesterol levels were not correlated with plasma total cholesterol levels measured at the same blood draw (*n* = 24). Furthermore, these associations of PCs with cellular cholesterol are opposite those with plasma cholesterol; PC1_CMTN_ was inversely (*r* = –0.21; *P* = 1.35 × 10^–10^) and PC2_CMTN_ was positively (*r* = 0.17; *P* = 1.89 × 10^–7^) correlated with plasma cholesterol levels (*n* = 635 with euglycemia).

To further examine whether effects of mRNA expression are likely mediated by corresponding changes in protein levels, we performed Western blot analysis of the same 24 MESA Exam 6 monocyte samples. Protein levels of ABCG1 were positively associated with its mRNA expression (*r* = 0.41; *P* = 0.04) and inversely associated with cellular cholesterol (*r* = –0.61; *P* = 0.002; Figure 4B), consistent with *ABCG1* mRNA findings. Although similar findings were observed for MYLIP, the association between its protein expression and mRNA expression was marginally significant (*r* = 0.39; *P* = 0.058), and the associations between protein levels and cellular cholesterol did not reach statistical significance (*P* = 0.22). The observed weak correlation between mRNA and protein associations is likely due to the semiquantitative method of Western blot analysis.

## Discussion

In the community-based MESA cohort free from prediabetes or T2D at baseline, we identified that expression of 3 LXR target genes, *ABCG1* and *ABCA1* (cholesterol efflux genes) and *MYLIP* (cholesterol uptake suppression gene), was inversely associated with incident prediabetes/T2D, independent of traditional risk factors. These findings were replicated using independent samples as well as more advanced sequencing technology for transcript quantification. These findings were robust in sensitivity analyses (e.g., controlling for LDL-C and fasting glucose, or limited to subjects with or without statin use), and were consistently observed across various age, sex, race and ethnicity, and overweight/obesity subgroups. PCA analyses showed that CMTN consists mainly of 2 independent cholesterol metabolism pathways, PC1_CMTN_ representing expression of SREBP2 target genes and PC2_CMTN_ representing expression of LXR target genes. Expressions of the 3 LXR target genes — *ABCG1*, *ABCA*, and *MYLIP* — were strongly correlated with each other and PC2_CMTN_; thus, their gene effects on prediabetes/T2D were not independent from each other, and can be represented by PC2_CMTN_, reflecting an aberrant LXR/ABCG1-ABCA1-MYLIP pathway (LAAMP) rather than an isolated effect of each gene alone.

Previously, we have reported that expression of 11 CMTN gene members was cross-sectionally associated with T2D prevalence (6), whereas the present study identified only 3 LXR target genes, *ABCA1*, *ABCG1*, and *MYLIP*, rather than SREBP2 target genes (cholesterol synthesis, e.g., *SQLE*, or uptake genes, e.g., *LDLR*), as associated with incident T2D. Since two decades ago, preclinical studies have also demonstrated that LXR agonists improve both insulin sensitivity and secretion while increasing expression of lipogenic enzymes and fatty acid synthase (13, 15, 16); but the precise mechanisms remain elusive, and little is known about the clinical relevance of the LXR signaling pathway in humans. Our data showed that, in addition to coexpressed *ABCG1*, *ABCA1*, and *MYLIP*, expression of *NR1H2* (encoding LXRβ) itself was also inversely associated with incident prediabetes/T2D, suggesting, for the first time to our knowledge, that dysregulated LAAMP is a major risk factor for T2D.

Mouse models disrupting cholesterol efflux have shown mixed results, suggesting that aberrations in *ABCA1* or *ABCG1* alone are not sufficient to cause T2D. For example, mice with pancreatic β cell–specific *Abca1* knockout (KO) showed accumulation of cholesterol in β cells and decreased insulin secretion (17–20); however, mice with global *Abca1* KO did not show alterations in insulin secretion or sensitivity (21, 22). This notion is further corroborated by human data showing that diabetes is not a characteristic feature of Tangier disease (homozygous loss-of-function variants in *ABCA1* gene) and by studies showing inconsistent association of *ABCA1* gene variation with T2D (18, 20, 22–24). Also, global *Abcg1* KO has small effects on insulin secretion and no effect on insulin sensitivity (18, 20). While overexpression of MYLIP (IDOL), which suppresses cellular cholesterol uptake, can raise plasma LDL levels in mice (12), its role in glucose metabolism has not been reported in preclinical studies. Here, we link dysregulated *ABCG1*/*ABCA1*/*MYLIP* at mRNA and protein levels to cellular cholesterol accumulation in human monocytes. Taken together with preclinical findings that cellular cholesterol accumulation causes pancreatic β cell dysfunction and insulin resistance (1–5, 25), our data provide what is to our knowledge the first evidence in humans supporting a role for dysregulated LAAMP and resultant cellular cholesterol accumulation in the pathogenesis of diabetes.

Consistent with clinical data that hypercholesterolemia is not a T2D risk factor, low or high circulating cholesterol per se is not likely to contribute to cellular cholesterol accumulation because of a cholesterol-mediated negative-feedback system that maintains cellular cholesterol homeostasis (26, 27). This negative-feedback system is regulated by SREBP2 and LXR, two transcription factors that maintain the balance of cellular cholesterol synthesis, uptake, and efflux, via regulation of CMTN. However, the negative-feedback regulation of cholesterol metabolism can be overridden, as shown by preclinical studies demonstrating that infection or inflammatory stress can lead to LAAMP dysregulation and increased intracellular cholesterol content in several cell types that are relevant to T2D and its complications, e.g., hepatocytes, vascular smooth muscle cells, human kidney mesangial cells, and macrophages (28–33). Cytokine administration reduces mRNA levels of *LXR*, as well as both protein and mRNA levels of retinoid X receptors (*RXR*s; LXR coregulators), thus inhibiting the LXR/ABCA1 pathway (30, 33). These data suggest that the mediating effects of the LAAMP on the diabetogenic effects of obesity that we observed may be related to elevated cytokines associated with obesity. Taken together, our data indicate that aberrant LAAMP and its resultant cellular cholesterol accumulation may, in part, underpin the effects of important T2D risk factors such as obesity or chronic inflammation.

Assessing the clinical relevance of the aberrant LAAMP and cellular cholesterol accumulation in human pancreatic β cell or skeletal muscle is practically challenging when large sample sizes are needed in observational studies. In this study, we used circulating monocytes, which are key cells of innate immunity and major contributors to the pathogenesis of inflammatory diseases including T2D (34). Risk prediction results similar to those using monocyte RNA-Seq data were also found using MESA RNA-Seq data from PBMCs (collected at Exam 5, *n* = 536; data not shown), suggesting that readily accessible blood cells may serve as a surrogate for insulin-sensitive cells in studying cholesterol metabolism — a mechanism critical to most living cells — and its clinical relevance to disease.

The increasing incidence of T2D and its macrovascular and microvascular complications constitute a major challenge to global health and underscore the need for better prevention and treatment of prediabetes and T2D. Current antidiabetic agents improve glycemia but have not effectively prevented diabetes complications. Ongoing development of new therapies for the prevention of prediabetes/T2D has not focused on cellular cholesterol metabolism (35, 36). In vitro, LXR agonists lead to net cellular cholesterol reduction (37). However, systemic LXR activation has adverse effects, such as hepatic steatosis, hypertriglyceridemia, and hypercholesterolemia (38), as a consequence of broad effects of LXR including induction of lipogenic genes. Development of LXR-selective agonists for diabetes treatment requires a better understanding of LXR antidiabetic mechanisms (39). Our data showed that expression of LXR/cellular cholesterol reduction pathway genes, not LXR/lipogenic pathway genes, was associated with incident prediabetes/T2D. Our findings, linking the aberrant LAAMP to cellular cholesterol accumulation and increased prediabetes/T2D risk, taken together with the emerging preclinical findings that cellular cholesterol is a reversible contributor to insulin resistance and β cell dysfunction (1–5), as well as nephropathy (40) and Alzheimer’s disease (41, 42), call for pharmacological targeting of the LAAMP to reverse cellular cholesterol accumulation for the prevention and treatment of T2D and its complications.

Several limitations of our study merit further comment. One limitation is potential residual confounding by adiposity or chronic inflammation in our multivariate models, even though we adjusted for BMI, plasma IL-6, and high-sensitivity C-reactive protein levels. Nonetheless, under the hypothesis, supported by in vitro and in vivo studies, that elevated levels of inflammatory biomarkers would disrupt the cholesterol-mediated feedback regulation (28–31), the effects of inflammatory biomarkers on T2D are likely mediated through the LAAMP. Our mediation analyses support the notion that expression of *LXR*/*ABCG1-ABCA1-MYLIP* is likely to be a mediator of adiposity measures or inflammatory biomarkers, and the latter are less likely to be confounders for the observed effects of the gene expression levels. Additionally, we used a single baseline measurement of CMTN gene expression, which may not fully reflect their cumulative effects.

To the best of our knowledge, our prospective cohort study revealed for the first time that the aberrant LAAMP is a powerful independent risk determinant for prediabetes/T2D in normal, overweight, and obese individuals, in both male and female and in White and African American individuals. Our data suggest the added value of measuring the LAAMP for early identification of individuals at high risk for T2D, especially those overweight, obese, or younger than 70 years. In conjunction with emerging experimental data, our epidemiological studies support a potential role for the LAAMP in T2D pathogenesis. Coupled with our study of correlation between LAAMP gene expression and cellular cholesterol levels, our data also support that cellular cholesterol accumulation, as found in circulating monocytes, may be a fundamental mechanism in the development of prediabetes and T2D. These findings support targeting of the LAAMP to reverse cellular cholesterol accumulation as a therapeutic strategy for prevention and treatment of T2D.

## Methods

### Sex as a biological variable.

All statistical analyses were adjusted for sex as a biological variable.

### Study participants.

The present prospective study (Figure 1) is primarily based on analyses of purified monocyte samples collected during the April 2010–February 2012 examination (Exam 5) of 934 MESA participants with euglycemia from 4 MESA sites (Johns Hopkins University, Columbia University, the University of Minnesota, and Wake Forest University).

### Blood collection and processing.

For the 1,349 MESA participants with euglycemia at Exam 5 and the 24 participants at Exam 6, blood was collected in sodium heparin–containing Vacutainer CPT cell separation tubes (Becton Dickinson) to separate PBMCs from other elements within 2 hours after blood draw. Subsequently, monocytes were purified on-site with anti-CD14 monoclonal antibody–coated magnetic beads, using an autoMACS automated magnetic separation unit (Miltenyi Biotec) as previously described (43). Purified monocytes were further processed using the AllPrep DNA/RNA/Protein Mini Kit (Qiagen, catalog 80004) on a QIAcube Connect MDx for downstream sequencing and Western blot assays.

### Transcriptomic profiling of monocytes using microarray technology.

For the Initial Study (Figure 1), global mRNA expression was quantified using Illumina microarray (HumanHT-12 v4 Expression) BeadChips as we previously described (43). For the Replication or Combined Study, mRNA sequencing using the method previously described (43) and total RNA sequencing (described below) were performed. The 24 monocyte samples purified at MESA Exam 6 also underwent total RNA sequencing.

### Transcriptomic profiling of monocytes by total RNA sequencing.

Ribosomal RNA was depleted, and strand-specific libraries were constructed using Illumina’s TruSeq Stranded Total RNA Library Prep Kit with Ribo-Zero Human/Mouse/Rat High Throughput kit (96 samples, 96 indexes) (Illumina, RS-122-2203). Two hundred fifty nanograms of total RNA was depleted of cytoplasmic rRNA and fragmented into smaller pieces (~140 nt). Cleaved RNA fragments were converted to first-strand cDNA using reverse transcriptase and random primers. Following Illumina’s standard protocol, the final cDNA library was created. The libraries were validated using Agilent TapeStation and quantitated using Quant-iT dsDNA HS Kit (Invitrogen) and quantitative PCR.

A set of 24 individually indexed cDNA libraries were pooled and sequenced on each lane of Illumina’s NovaSeq S4 flow cell to get a minimum of 100 million reads per sample. The libraries were clustered and sequenced using NovaSeq 5000/6000 S4 Reagent Kit (300 cycles) (cat. 200012866) to 2 × 151 cycles. Illumina NovaSeq Control Software v1.3 was used to provide the management and execution of the NovaSeq 6000 and to generate BCL files. The BCL files were converted to FASTQ files, adapters were trimmed off, and reads were demultiplexed using bcl2fastq Conversion Software v2.20.

The FASTQ files were trimmed using the *fastp* preprocessing tool (44). Reads were aligned to the human genome and transcriptome using the current version of STAR (https://github.com/alexdobin/STAR?tab=readme-ov-file) with the 2-pass option to allow for the identification of novel exon-exon junctions. We performed quality control at the level of the raw and trimmed FASTQ files with at least 10 reads in one sample and at the sample level (present in ≥90% of the samples).

### Combining mRNA-Seq data from mRNA and total RNA sequencing data.

mRNA and total RNA sequencing data (raw counts) from Exam 5 were combined using ComBat-seq (45) to remove batch effects. Normalization between samples was performed using the trimmed mean of M-values (TMM) normalization method (46). To be able to continue to use the flexible and computationally efficient linear modeling functions in R, we applied the *voom* transformation implemented in the *voom* function of the *limma* R package (47), which transforms the raw count data to log_2_ counts per million (*y* = logCPM) and provides weights to account for residual variance heterogeneity. Flow cell effects were included in the models or removed.

### Monocyte CMTN quantification.

Monocyte mRNA expression data for the 11 CMTN gene members, *ABCG1* and *ABCA1* (cholesterol efflux), *LDLR* and *MYLIP* (uptake), and *SCD*, *FADS1*, *HMGCS1*, *FDFT1*, *SQLE*, *CYP51A1*, and *SC4MOL* (synthesis), as well as *NR1H3* and *NR1H2* (encoding LXRα and LXRβ, respectively) and their lipogenic LXR target genes (14), were extracted from the microarray or mRNA-Seq data sets.

### Cellular cholesterol measurements by gas chromatography.

Cellular cholesterol levels were measured using the gas chromatography (GC) method (48) for the 24 randomly selected monocyte samples purified at MESA Exam 6. Around 1 million cells were used for cholesterol extraction. After spin-down and removal of the supernatant (PBS), 1 mL hexane containing 2 μg of 5α-cholestane (internal standard) was added to the cell pellet. The sample was heated at 60°C for an hour to extract cholesterol. After centrifugation, the cell debris was used for total protein quantification by bicinchoninic acid (BCA) protein assay, and the hexane phase was transferred into a test tube to dry down under nitrogen. Dried residue was resuspended in 0.2 mL hexane and injected onto the GC column for free cholesterol measurement. Then the hexane phase was completely transferred from the GC vial to a round-bottom screw-cap tube. After hexane dry-off, 1 mL of 95% ethanol and 0.1 mL of 50% KOH were added and mixed by vortexing. The sample was heated at 60°C for an hour with vortexing every 20 minutes. Then, 1 mL water and 1 mL hexane were added with vortex mixing by low-speed centrifugation for 5 minutes to separate the 2 phases. Afterward, the hexane phase was removed and remaining residue dried under nitrogen. The residue was resuspended in 0.2 mL hexane and injected onto a GC for total cholesterol measurement. Esterified cholesterol was calculated as total minus free cholesterol. Total protein amount, determined by BCA assay, was used for normalization of cholesterol values.

### Western blot analysis and protein quantification.

Isolated proteins were resolubilized in 2.5% SDS solution and protein concentrations determined using the BCA Protein Assay Kit (Millipore, catalog 71285-3). An equal amount of total protein (10 μg per lane) and Precision Plus Protein Dual Color Standard (Bio-Rad, catalog 1610374) was loaded on 4%–20% Mini-PROTEAN TGX Stain-Free Protein gels (Bio-Rad, catalog 4568094), resolved by SDS-PAGE electrophoresis, and then transferred onto 0.45 μm PVDF membrane (Bio-Rad, catalog 1704275). After transfer, membrane was cut horizontally at 80 kDa and 150 kDa to detect proteins below 80 kDa, between 80 and 150 kDa, and above 150 kDa. After blocking in 5% blocking buffer (Bio-Rad, catalog 1706404) for 1 hour at room temperature, the membranes were incubated with primary antibodies in 3% BSA overnight at 4°C: rabbit anti-ABCG1 (Abcam, catalog ab52617; diluted 1:1,000), rabbit anti-MYLIP (Invitrogen, catalog PA5-96524; diluted 1:500), mouse anti-ABCA1 (Abcam, catalog ab1818; diluted 1:200), and mouse anti–β-actin (Sigma-Aldrich, catalog A5441; diluted 1:10,000). This was followed by appropriate HRP-conjugated goat anti-rabbit IgG (Abcam, catalog ab205718) or goat anti-mouse IgG (Abcam, catalog ab205719) incubation for 1 hour at room temperature. Protein signals were activated using SuperSignal West Pico PLUS Chemiluminescent Substrate (Thermo Fisher Scientific, catalog 34580) and imaged on the Bio-Rad ChemiDoc MP Imaging system (Supplemental Figure 2). Protein levels were log_2_ plus 1 transformed in the analyses as mRNA expression levels.

### Definition of obesity, prediabetes, and T2D.

Weight was measured with a Detecto Platform Balance Scale to the nearest 0.5 kg. Height was measured with a stadiometer (Accu-Hite Measure Device with level bubble) to the nearest 0.1 cm. Body mass index (BMI) was defined as weight in kilograms divided by square of height in meters (kg/m^2^). Individuals with a BMI of 30 kg/m^2^ or more were considered to have obesity, and those with a BMI of 25–29.9 kg/m^2^ were considered overweight. Fasting serum glucose at each examination was measured by rate reflectance spectrophotometry using thin-film adaptation of the glucose oxidase method on a Vitros analyzer (Johnson & Johnson Clinical Diagnostics). Prediabetes was defined as fasting glucose 100–125 mg/dL (without anti-diabetes medication use), T2D as fasting glucose greater than 125 mg/dL or anti-diabetes medication used.

### Statistics.

CMTN (11 members) was analyzed at an individual gene level as well as by a principal component analysis (PCA) capturing the independent sets of cholesterol metabolism pathways in the data. Before PCA, variables were scaled to unit variance. The discrete time proportional hazards model was used to examine the predictive effects of CMTN on incident prediabetes and diabetes. In the full model, covariates included age, sex, race and ethnicity, cigarette smoking, physical activity level, BMI, plasma triglycerides, and high-density lipoprotein cholesterol (HDL-C). Sensitivity analyses included additional covariates, such as low-density lipoprotein cholesterol (LDL-C), total cholesterol, systolic blood pressure, fasting glucose levels, statin use, and prevalent CVD at Exam 5, as well as plasma interleukin-6 (IL-6) (49) and high-sensitivity C-reactive protein (hsCRP) (50) measured at Exam 1 (10 years prior, the only Exam with IL-6 and hsCRP available). To compare categorical variables across subgroups, χ^2^ test was used. Pearson’s correlation coefficient was used to assess correlations between continuous variables.

The contribution of CMTN in the prediction of prediabetes/T2D, over and above that of traditional risk factors, was analyzed with the use of multiple discrimination and reclassification indexes (51), including Harrell’s C statistic (52), likelihood ratio test, and the net reclassification improvement index (NRI) (53). The mediation analyses were performed by structural equation modeling using robust (against deviations from normality) methods for computing standard errors, test statistics, and confidence intervals (54).

Two-sided *P* values less than 0.05 were considered to indicate statistical significance. All the analyses were conducted on measurements that were taken from distinct samples or substudies. Statistical analyses were performed in R v4.1.0 (http://www.r-project.org/) using the following packages: stats v4.1.0, survival v3.2-13, lattice v0.20-45, lavaan v0.6-11, pROC v1.18.0, and nricens v1.6.

### Study approval.

The MESA study protocol was approved by the Institutional Review Board at each site (Johns Hopkins University, Baltimore, Maryland, USA; Columbia University, New York, New York, USA; University of Minnesota, Minneapolis, Minnesota, USA; and Wake Forest University, Winston-Salem, North Carolina, USA.) (https://clinicaltrials.gov/study/NCT00005487). All participants signed informed consent. The present study was determined exempt and approved by the Duke Institutional Review Board (protocol Pro00102902).

### Data availability.

Data used in this study can be obtained through NCBI’s Gene Expression Omnibus (GEO GSE56046), the database of Genotypes and Phenotypes (dbGaP phs000209 [MESA cohort]), and the MESA Data Coordinating Center (https://www.mesa-nhlbi.org/Publications.aspx). Values for all data points in graphs are reported in the Supporting Data Values file.

## Author contributions

All authors contributed to data curation, formal analysis, and critical revision of the manuscript, including JD, ATN, KL, MTH, DP, LH, JT, DV, JKS, EB, MPB, AB, JSP, HIR, MOG, RPT, DMM, DAD, SSR, BMP, DS, CBN, DH, IH, SSR, JHS, MPB, WP, DJ, JSP, and YL. YL, JD, KL, DJ, and IH contributed to statistical analysis and methodology. YL, JD, ATN, and KL contributed to study investigation, project administration, and validation. YL, JD, and ATN contributed to conceptualization, visualization, and original draft writing. YL and JD contributed to supervision and funding acquisition.

## Supplementary Material

Supplemental data

ICMJE disclosure forms

Supporting data values

## Figures and Tables

**Table 2:**
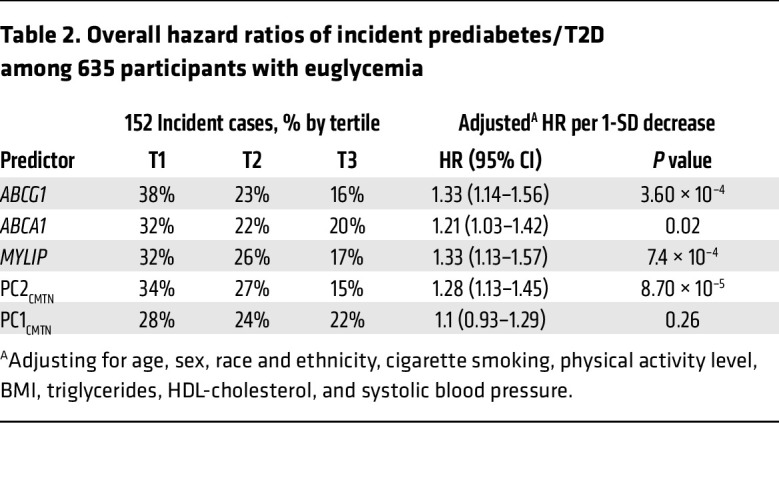
Overall hazard ratios of incident prediabetes/T2D among 635 participants with euglycemia

**Table 1:**
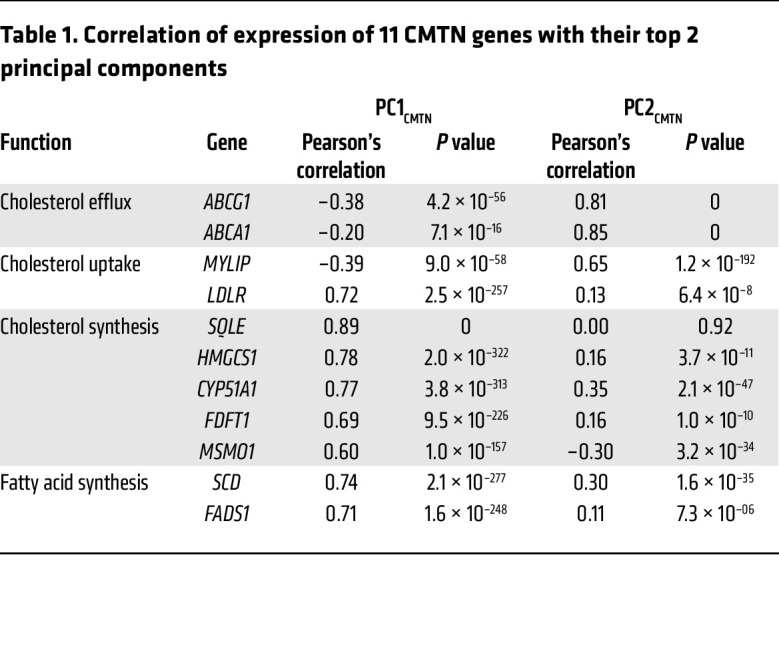
Correlation of expression of 11 CMTN genes with their top 2 principal components

## Version 1 05/15/2024

Electronic publication

## Version 2 07/30/2024

Added omitted author Daniel Duprez. See related corrigendum published August 15, 2024.
