# LXR signaling pathways link cholesterol metabolism with risk for prediabetes and diabetes

**DOI:** 10.1172/JCI185290

**Published:** 2024-08-15

**Authors:** Jingzhong Ding, Anh Tram Nguyen, Kurt Lohman, Michael T. Hensley, Daniel Parker, Li Hou, Jackson Taylor, Deepak Voora, Janet K. Sawyer, Elena Boudyguina, Michael P. Bancks, Alain Bertoni, James S. Pankow, Jerome I. Rotter, Mark O. Goodarzi, Russell P. Tracy, David M. Murdoch, Daniel Duprez, Stephen S. Rich, Bruce M. Psaty, David Siscovick, Christopher B. Newgard, David Herrington, Ina Hoeschele, Steven Shea, James H. Stein, Manesh Patel, Wendy Post, David Jacobs, John S. Parks, Yongmei Liu

Original citation: *J Clin Invest*. 2024;134(10):e173278. https://doi.org/10.1172/JCI173278

Citation for this corrigendum: *J Clin Invest*. 2024;134(16):e185290. https://doi.org/10.1172/JCI185290

During the preparation of this manuscript, Daniel Duprez’s information was inadvertently omitted from the author list and the *Author contributions* section of the manuscript. Christopher B. Newgard’s middle initial was also omitted from both. The correct author list, affiliation list, and sentence from the *Author contributions* section are below. The HTML and PDF versions have been updated.

Jingzhong Ding,^1^ Anh Tram Nguyen,^2^ Kurt Lohman,^2^ Michael T. Hensley,^2^ Daniel Parker,^3^ Li Hou,^2^ Jackson Taylor,^4^ Deepak Voora,^2^ Janet K. Sawyer,^1^ Elena Boudyguina,^1^ Michael P. Bancks,^5^ Alain Bertoni,^1^ James S. Pankow,^6^ Jerome I. Rotter,^7^ Mark O. Goodarzi,^8^ Russell P. Tracy,^9^ David M. Murdoch,^10^ Daniel Duprez,^11^ Stephen S. Rich,^12^ Bruce M. Psaty,^13^ David Siscovick,^14^ Christopher B. Newgard,^15^ David Herrington,^1^ Ina Hoeschele,^16^ Steven Shea,^17^ James H. Stein,^18^ Manesh Patel,^2^ Wendy Post,^19^ David Jacobs Jr.,^6^ John S. Parks,^1^ and Yongmei Liu^2^

^1^Department of Internal Medicine, Wake Forest School of Medicine, Winston-Salem, North Carolina, USA. ^2^Department of Medicine, Division of Cardiology, and ^3^Department of Medicine, Division of Geriatrics, Duke University, Durham, North Carolina, USA. ^4^Department of Biological, Geological, and Environmental Sciences, Cleveland State University, Cleveland, Ohio, USA. ^5^Department of Epidemiology and Prevention, Wake Forest School of Medicine, Winston-Salem, North Carolina, USA ^6^Division of Epidemiology and Community Health, University of Minnesota, Minneapolis, Minnesota, USA. ^7^The Institute for Translational Genomics and Population Sciences, Department of Pediatrics, The Lundquist Institute for Biomedical Innovation at Harbor-UCLA Medical Center, Torrance, California, USA ^8^Division of Endocrinology, Diabetes and Metabolism, Cedars-Sinai Medical Center, Los Angeles, California, USA. ^9^Department of Pathology and Laboratory Medicine, University of Vermont, Burlington, Vermont, USA. ^10^Department of Medicine, Division of Pulmonary, Allergy, and Critical Care Medicine, Duke University, Durham, North Carolina, USA. ^11^Cardiovascular Division, Department of Medicine, University of Minnesota, Minneapolis, Minnesota, USA. ^12^Center for Public Health Genomics, University of Virginia, Charlottesville, Virginia, USA. ^13^Cardiovascular Health Research Unit, Departments of Medicine, Epidemiology, and Health Systems and Population Health, University of Washington, Seattle, Washington, USA. ^14^New York Academy of Medicine, New York, New York, USA. ^15^Department of Pharmacology and Cancer Biology, Duke University, Durham, North Carolina, USA. ^16^Fralin Life Sciences Institute, Virginia Tech, Blacksburg, Virginia, USA. ^17^Department of Medicine, Columbia University, New York, New York, USA. ^18^School of Medicine and Public Health, University of Wisconsin, Madison, Wisconsin, USA. ^19^Division of Cardiology, Department of Medicine, Johns Hopkins University, Baltimore, Maryland, USA. 

Author contributions

JD, ATN, KL, MTH, DP, LH, JT, DV, JKS, EB, MPB, AB, JSP, HIR, MOG, RPT, DMM, DAD, SSR, BMP, DS, CBN, DH, IH, SSR, JHS, MPB, WP, DJ, JSP, and YL contributed to data curation, formal analysis, and critical revision of the manuscript.

The authors regret the errors.

## Version 1 08/15/2024

Electronic publication

