# Supplementary material for: LXR signaling pathways link cholesterol metabolism with risk for prediabetes and diabetes
Source: J Clin Invest. 2024 May 15;134(10):e173278. doi: 10.1172/JCI173278 (PMC11093600; doi:10.1172/JCI173278)
Supplement: Supplemental data [file jci-134-173278-s007.pdf]

Supplemental Table 1. Baseline characteristics of the 1,349 MESA participants.

**Characteristic**	All* (n=1,349)
Age, yr	69 (9)
Sex	
Male	42%
Female	58%
Race/Ethnicity	
White	55%
African American	26%
Hispanic or Latino	19%
Cigarette Smoking	
Former Smoker	50%
Current smoker	9%
Physical Activity (MET-hr/wk)	3056 (3773)
BMI, kg/m^2	28 (5)
Triglycerides, mg/dL	99 (48)
LDL-C, mg/dL	109 (32)
HDL-C, mg/dL	58 (17)
SBP, mmHg	123 (20)
Fasting Glucose, mg/dL	90 (6)
Prevalent CVD	5%
Statin use	32%

*Mean (SD) provided for continuous variables.

Supplemental Table 2. Pairwise Correlation Between Expression of Three LXR Target Genes in the Initial Study (blue) and Replication Study (orange).

	ABCG1	*ABCA1*	*MYLIP*
ABCG1		0.71	0.47
ABCA1	0.68		0.52
MYLIP	0.63	0.47	

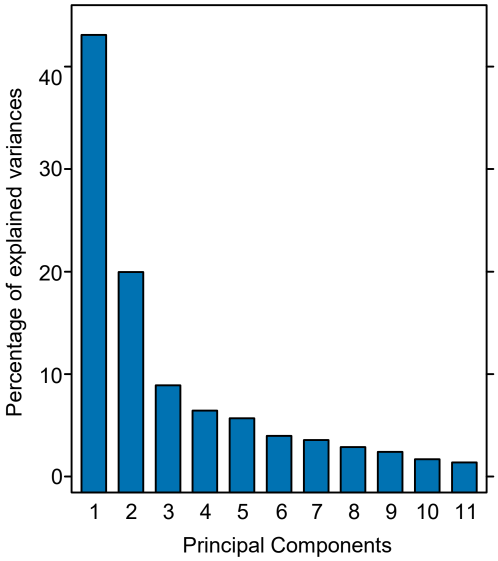

Supplemental Figure 1. CMTN principal components analysis of 11 CMTN gene members for all 635 participants with euglycemia and RNA-seq data. Shown is a Scree plot of percentage of explained variance for each PC.

Supplemental Table 3. Hazard ratios of incident prediabetes/T2D among 635 participants with euglycemia. Stratified by age, sex, and race/ethnicity.

	Incident cases, % by tertile				Adjusted* HR (95% CI) per 1-SD decrease						
	All (n=635)				Age, yr		Sex		Race/ethnicity		
					<70 n=390	≥70 n=245	Female n=370	Male n=265	Caucasian n=356	AA n=174	Hispanics n=105
**Predictor**	**T1**	**T2**	**T3**	**# Incident cases**	**(89)**	**(63)**	**(74)**	**(78)**	**(71)**	**(52)**	**(29)**
ABCG1	38%	23%	16%		1.5 (1.17-1.93)	1.28 (1.03-1.60)	1.41 (1.16-1.71)	1.27 (0.99-1.64)	1.42 (1.09-1.84)	1.38 (1.10-1.73)	0.92 (0.59-1.44)
ABCA1	32%	22%	20%		1.35 (1.07-1.71)	1.08 (0.85-1.37)	1.21 (0.95-1.54)	1.24 (0.98-1.55)	1.38 (1.09-1.76)	1.15 (0.87-1.51)	0.9 (0.57-1.41)
MYLIP	32%	26%	17%		1.4 (1.10-1.80)	1.26 (0.99-1.61)	1.22 (0.97-1.52)	1.53 (1.18-2.00)	1.28 (0.97-1.67)	1.39 (1.11-1.74)	1.25 (0.77-2.04)
$PC2_{CMTN}$	34%	27%	15%		1.55 (1.24-1.94)	1.16 (0.98-1.39)	1.29 (1.10-1.50)	1.29 (1.05-1.58)	1.36 (1.08-1.70)	1.27 (1.08-1.49)	0.92 (0.53-1.58)
$PC1_{CMTN}$	28%	24%	22%		1.11 (0.86-1.45)	1.05 (0.85-1.29)	1.27 (0.99-1.64)	0.97 (0.77-1.21)	0.92 (0.71-1.20)	1.21 (0.97-1.50)	1.11 (0.68-1.81)

Supplemental Table 4. Hazard Ratios of Incident Prediabetes/T2D among 635 Participants with Euglycemia.

**Gene**	**Tertile**	**Hazard Ratio (95%, CI)**	***p* value**
HMGCS1	1	ref.	
	2	0.98 (0.66-1.45)	0.91
	3	0.85 (0.56-1.28)	0.42
SQLE	1	ref.	
	2	0.98 (0.66-1.46)	0.94
	3	0.79 (0.51-1.22)	0.29
SCD	1	ref.	
	2	0.80 (0.55-1.19)	0.27
	3	0.73 (0.48-1.10)	0.13
MSMO1	1	ref.	
	2	1.04 (0.70-1.54)	0.83
	3	0.99 (0.64-1.53)	0.97
FDF	1	ref.	
	2	0.96 (0.64-1.43)	0.83
	3	1.08 (0.72-1.61)	0.70
FADS1	1	ref.	
	2	0.73 (0.49-1.08)	0.11
	3	0.84 (0.56-1.24)	0.38
CYP51A1	1	ref.	
	2	1.20 (0.81-1.78)	0.37
	3	0.94 (0.61-1.46)	0.79
LDLR	1	ref.	
	2	0.92 (0.63-1.34)	0.66
	3	0.72 (0.47-1.10)	0.13

Supplemental Table 5. Baseline Characteristics of 635 MESA Participants with Euglycemia and Its Correlation with *ABCG1* Expression.

Characteristic*	*ABCG1*			Correlation with *ABCG1* expression†	*P* value
	Tertile 1 (n=166)	Tertile 2 (n=208)	Tertile 3 (n=261)		
Age, yr	68.6 (8.8)	67.6 (8.6)	65.9 (8.1)	-0.16	5.1E-05
Sex (%)					0.35
Male	37	45	42		
Female	63	55	58		
Race/Ethnicity (%)					0.02
White	50	55	62		
African American	36	28	21		
Hispanic or Latino	14	17	17		
Cigarette Smoking (%)					0.60
Former Smoker	53	47	47		
Current smoker	8	9	11		
Physical Activity (MET-hr/wk)	3454 (4248)	3227 (3820)	3568 (4293)	0.02	0.55
BMI, kg/m^2	29 (5)	28 (5)	27 (5)	-0.21	1.0E-07
Triglycerides, mg/dL	111 (58)	101 (44)	91 (49)	-0.19	2.5E-06
LDL-C, mg/dL	104 (30)	111 (30)	117 (31)	0.21	8.7E-08
HDL-C, mg/dL	54 (16)	57 (15)	61 (18)	0.20	2.7E-07
SBP, mmHg	123 (19)	121 (20)	120 (19)	-0.06	0.12
Fasting Glucose, mg/dL	90 (6)	90 (6)	89 (6)	-0.10	0.01
Prevalent CVD (%)	6	6	2		0.04
Statin use (%)	46	40	19		1.2E-09

*Mean (SD) provided for continuous variables; †for continuous variables.

Supplemental Table 6. Correlations* between expression of CMTN genes and cholesterol levels in 24 primary monocyte samples.

		Total Cholesterol		Free Cholesterol		Esterified Cholesterol	
**Function**	**Gene**	***r***	***p* value**	***r***	***p* value**	***r***	***p* value**
Cholesterol efflux	*ABCG1*	-0.69	2.3E-04	-0.53	0.02	-0.64	0.002
	ABCA1	-0.41	0.07	-0.46	0.04	-0.21	0.38
Cholesterol uptake	*MYLIP*	-0.35	0.14	-0.34	0.14	-0.22	0.35
	LDLR	0.48	0.03	0.54	0.01	0.25	0.29
Cholesterol synthesis	*SQLE*	0.40	0.08	0.58	0.01	0.08	0.74
	HMGCS1	0.25	0.30	0.45	0.04	-0.04	0.86
	CYP51A1	0.27	0.24	0.55	0.01	-0.08	0.74
	FDFT1	0.15	0.53	0.27	0.25	-0.02	0.93
	MSMO1	0.47	0.04	0.43	0.06	0.32	0.17
Fatty acid synthesis	*SCD*	0.00	0.99	0.22	0.35	-0.19	0.41
	FADS1	0.22	0.36	0.30	0.20	0.06	0.80

*Age, sex, and race/ethnicity adjusted.

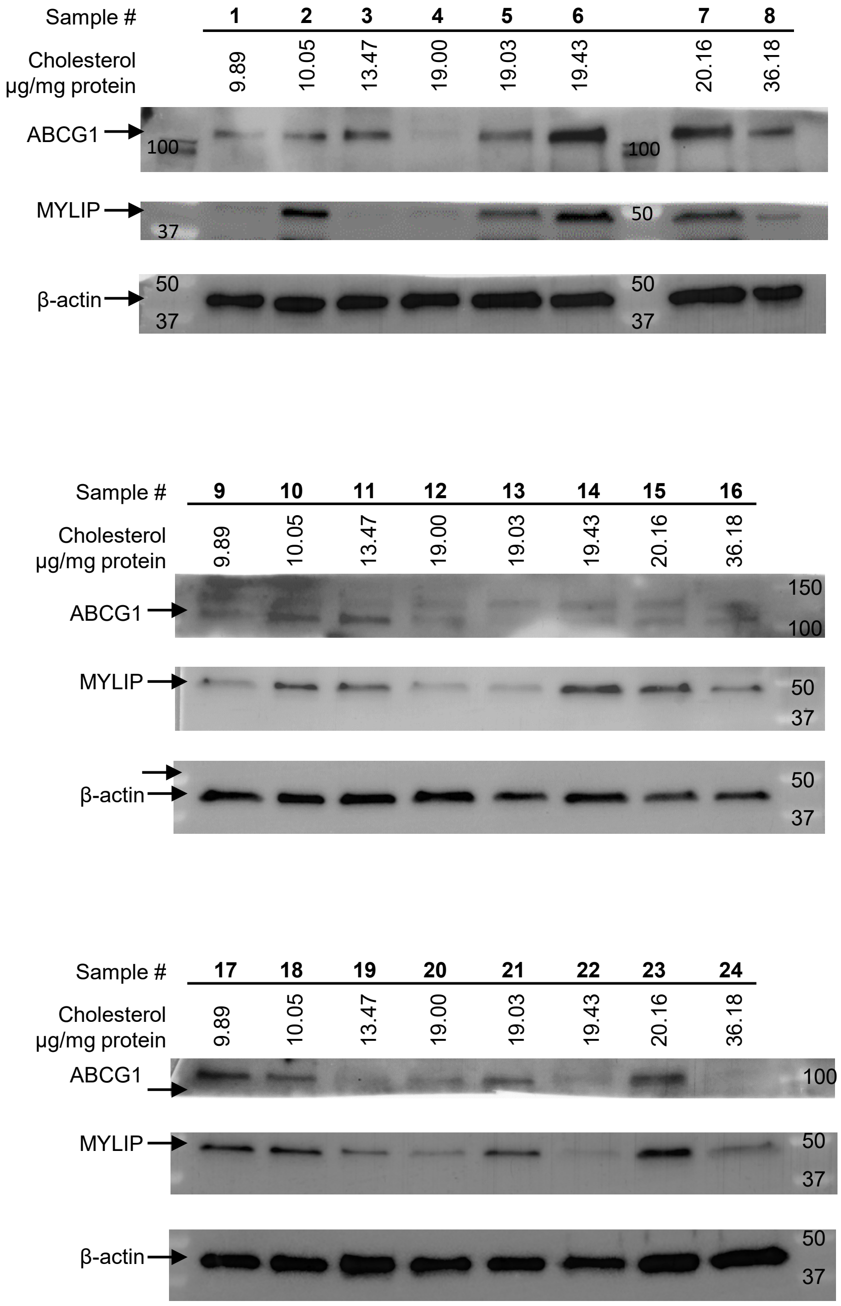

Supplemental Figure 2. Shown are Western Blot images of protein extracts from the 24 randomly selected monocytes. Membranes were cut and probed with primary antibodies for ABCG1, MYLIP, and β-actin. Corresponding total cholesterol values (µg/mg) are indicated.
